# The Possibility of Using Waste Phosphates from the Production of Polyols for Fertilizing Purposes

**DOI:** 10.3390/molecules27175632

**Published:** 2022-09-01

**Authors:** Paulina Bogusz

**Affiliations:** 1 Fertilizers Research Group, Łukasiewicz Research Network–New Chemical Syntheses Institute, Al. Tysiąclecia Państwa Polskiego 13a, 24-110 Puławy, Poland; paulina.bogusz@ins.lukasiewicz.gov.pl; 2 Department of Agricultural and Environmental Chemistry, University of Life Sciences in Lublin, Akademicka 13, 20-950 Lublin, Poland

**Keywords:** phosphorus, critical raw materials, circular economy, fertilizers, waste management, liquid waste

## Abstract

The limited resources of phosphorus raw materials, which are located outside of Europe, make it necessary to obtain phosphorus from the waste stream. This is reflected in the new EU Regulation 2019/1009, which concerns making fertilizing products available on the market. Its main goal is to harmonize the standards for fertilizers obtained from organic or secondary raw materials in the EU and to create new opportunities for their production and sale. The fertilizer sector uses almost 90% of the phosphorus raw material, for which there is no substitute, and the demand for fertilizers is constantly growing, stimulated by the growing number of people. A substitute for expensive imported raw materials can be waste sodium–potassium phosphate from the production of polyols. This study analyzes the composition and form of waste from PCC Rokita S. A. from Brzeg Dolny in Poland, in terms of the possibility of using it in the production of fertilizers according to the new EU regulations. Research shows that it contains nearly 19% phosphorus, as well as potassium and sodium. Low-level impurities and low organic matter content classify it as a raw material for the production of inorganic fertilizers. Due to the high water content, the most advantageous form of its use is the production of fertilizers in the form of a suspension. A certain difficulty in the production of this type of fertilizer may be the layers of crystals of different sizes precipitating in the waste.

## 1. Introduction

The population is growing very fast and in order to meet its requirements, plant growth intensification measures are necessary. Phosphorus, along with nitrogen and potassium, plays a key role here. As a building block of living organisms, it is one of the most important elements for plants and animals [1,2,3,4]. It is necessary to supplement its deficiencies in the soil [2,5].

Phosphorus deficiency in soil is often the main factor limiting plant growth. Its insufficient amount, especially in the early stages of the development of one-year-old plants, may result in a reduction in the yield. Later adjustment of its dose will not restore normal plant growth [6].

The world’s profitable reserves of phosphate are gradually declining [2]. It is estimated that their production capacity is in the range of 165–195 million tons per year. Specialists determine the duration of use of such resources differently, but they agree on one thing: they are a non-renewable source and sooner or later they will be exhausted [7,8]. Another problematic issue is the uneven distribution of phosphate mines of economic importance in the world [4,7]. More than two-thirds of the raw material they extract comes from Morocco, China, and the United States. Europe is almost entirely dependent on its imports [2,4,7,8].

The importance of this raw material for agricultural production is evidenced by the fact that over 80% of its resources are used for fertilization, where it is the basis for the production of almost all phosphorus fertilizers [7]. There is no way to compensate for its shortcomings in this sector by shifting resources from other industries [4,8].

This is particularly important due to the growing demand for fertilizers, stimulated by the continuous growth of the population [9]. The risk of deficits in the fertilizer market determines the threat to food production [10,11].

Rational phosphorus management is particularly important for European countries, where economically profitable resources of this raw material are small. As a result, Europe’s dependence on the import of this raw material reaches 90% [4,10,12]. In order to minimize the impact of external markets, European Union countries turned to the search for alternative sources of phosphorus, including those from waste materials. This corresponds to the goals of the circular economy, which assumes the minimization and recycling of waste and the extension of the life cycle of economic products [4,13,14,15].

Losses of phosphorus concern not only the waste of natural resources, but also water, energy, and other resources used in its processing cycle. Thus, the production of fertilizers powered by waste materials brings both ecological and economic benefits. Taking this course of action in the long term will enable the closure of the phosphorus cycle [4,16].

The distribution and availability of phosphate ores are not the only problems for the fertilizer industry and its beneficiary, the agricultural sector. The pollution associated with phosphate deposits is of great concern. Cadmium is particularly dangerous, as it is mainly contaminated with phosphate minerals of sedimentary origin. This applies to deposits located in North and East Africa and the Middle East. Less pollution is observed in minerals of volcanic origin (deposits in Finland, Russia, and South Africa). When it enters the soil through fertilizers, it easily migrates and accumulates in plants [4,10]. Some crops, such as tobacco, rapeseed, and sunflower, tend to take up cadmium in particularly large amounts [4]. In turn, consumption of contaminated crops is the main route of exposure for humans [10].

The bad news is that there is no substitute for phosphorus, which is a non-renewable resource; the good news is that phosphorus does not disappear after use. The basic scenario assumes its uptake from ore and dispersion in the environment as a result of fertilization. The key to maintaining the continuity of its flow is sustainable management and its effective recovery from the waste stream. This is the way to guarantee phosphorus resources for future generations [4,9].

Additionally, in order to emphasize high economic importance and high supply risk, phosphorus and phosphate rock were included in the list of critical natural resources [10].

Limited resources of phosphorus raw materials, the political situation in the areas of their extraction, poor quality of exploited deposits, and increasing requirements for fertilizer products based on these conditions necessitate the development of new technologies for the processing of waste phosphorus raw materials [2,4]

In the EU, there is a great potential for recovering phosphorus raw materials from waste materials, including: municipal and industrial wastewater, sewage sludge, ashes from sewage sludge incineration, meat and bone meal, slurry, biomass, and industrial waste. Intensive work is still underway to allow for the economic processing of this waste, mainly for fertilization purposes, where the demand for this element is the highest [17].

Many effective attempts to manage waste from various industries in the production of suspension fertilizers have been made. One of them is the development of sludge from the production of firefighting agents formed during alkaline hydrolysis of keratin, which is rich in phosphorus, potassium, and calcium [18]. Peat slime from the alkaline extraction process of humus compounds used to produce drugs and peat preparations is also successfully used [18]. For fertilizer purposes, breeding waste, such as slurry from poultry farms, can also be used [18]. The possibility of processing this into fertilizer in the form of a suspension of filtration slime from the production of extraction phosphoric acid was also tested [19,20]. The production of magnesium sulfate, consisting of the decay of roasted magnesite with sulfuric acid, is accompanied by the formation of sediment. It is a valuable source of calcium, magnesium, and sulfur, which can be successfully used in the production of suspension fertilizers [21]. Effective use of sodium sulfate solution, emerging as a by-product in the production of trimethylpropane, has also been confirmed [22]. An interesting proposition is the use of potassium waste sulfate from biofuels. There is no need for the application of an additional substance stabilizing the suspension, and there is a large possibility of configuring with other raw materials [23].

Recently, an interesting option, using waste from the production of polyols in the form of an aqueous suspension of sodium and potassium phosphates, has emerged. Due to the high water content of these wastes, the possibilities of their economic processing are limited and so far, they have not been used. The greater emphasis on the recovery of the raw material from the waste stream contained in the new EU fertilizer regulations has contributed to the review of the possibility of its use and is undergoing research with its use in the production of suspension fertilizers [18,19].

In Poland, the largest producer of polyols is PCC Rokita in Brzeg Dolny, which generates approximately 1500 tons/year of waste phosphate salts in the form of a liquid suspension. It cannot be returned to the process and reused.

The aim of the research is to assess the possibility of using waste phosphates from the production of polyols from PCC Rokita S. A. for fertilization purposes based on the physico-chemical analysis of the waste.

## 2. Results and Discussion

### 2.1. Organic Carbon Content Analysis Results

The waste was left for the time needed to stabilize it, and then six samples were taken from the surface layer and subjected to TOC analysis to determine whether an organic layer was separating on the surface. The obtained results were then compared with the results obtained for the mean sample. Since the waste is in the form of an unstable suspension (part of the solid phase falls to the bottom during storage), three samples were taken for the analysis of the averaged sample during intensive mixing with a stream of compressed air. The results of the TOC measurements are summarized in Table 1 for the surface layer and Table 2 for a homogeneous sample.

The samples taken from the surface layer showed a large variety of the obtained results. The results of the averaged samples were more stable. The average value of the organic carbon content in the surface layer (341 ppm) is lower than the average TOC value obtained for the homogenized sample (586 ppm). On this basis, it can be concluded that no organic layer is separated on the surface of the waste after settling. The mean TOC value of 586 ppm (0.0586%) in a sample homogenized by mixing with compressed air is low. Therefore, for a fertilizer produced by mixing this waste with other mineral raw materials, the organic carbon content will be even lower. According to the EU Regulation 2019/1009, a fertilizer with an organic carbon content below 3% (m/m) is classified as an inorganic fertilizer. The content of organic carbon at a level lower than 1% (m/m) additionally eliminates the need to test the content of pathogens in the fertilizer (*Salmonella spp. and Escherichia coli or Enterococcaceae*) [24].

### 2.2. The Results of the Chemical Analysis of the Waste

Waste phosphate from the production of polyols contains a large amount of water; therefore, it will be most economical to use it for the production of suspension fertilizers [25]. Therefore, Table 3 summarizes the results of the chemical analysis of waste along with the requirements for this type of fertilizer provided for in the new Regulation of the European Parliament and of the Council (EU) 2019/1009 of 5 June 2019: PFC 1 (C)(I)(b)(ii): multi-component liquid inorganic macronutrient fertilizer [24].

The high content of phosphorus in the waste, at the level of almost 19%, makes it an attractive raw material for fertilizing and confirms the economics of its use in the production of fertilizers. The content of pollutants in the waste is well below the threshold value established by the European Commission in the new regulations. This makes the tested waste a competitive raw material in relation to phosphate rock, often contaminated with cadmium [10,24].

### 2.3. The Results of the Sediment Composition Analysis in the Waste

**Figure 1:**
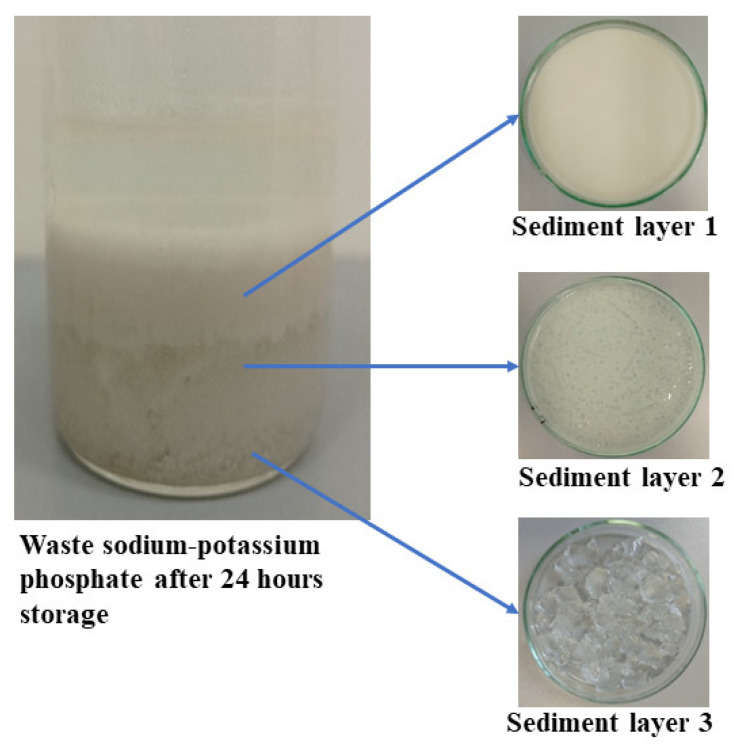
Sludge layers in waste phosphate with different crystal sizes.

During the storage of waste phosphate, three clearly separated layers are formed at its bottom (Figure 1). They differ in the composition and size of the crystals. The size of the crystals formed in the sediment is important when applying fertilizer based on waste phosphate from the production of polyols. The fine crystals that settle to the bottom can be successfully mixed again. On the other hand, large crystals will clog the spray nozzles in application/dispensing devices [25].

**Figure 2:**
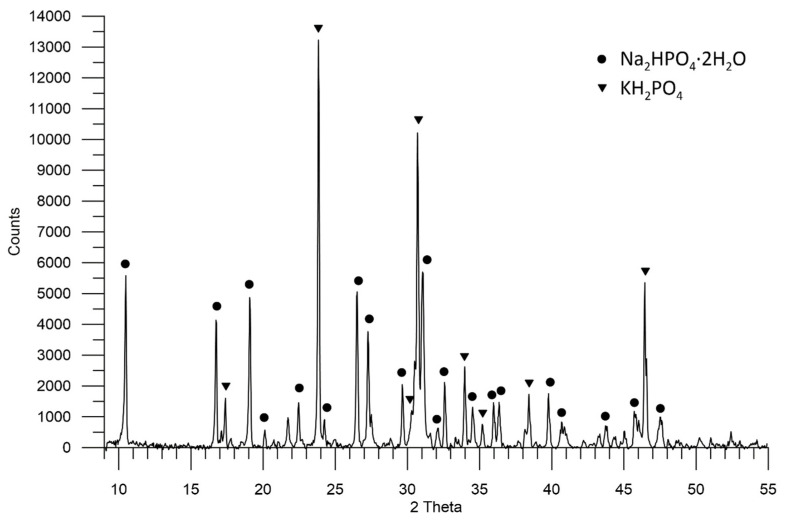
A diffractogram showing the composition of sediment 1 in the sample of waste phosphate from polyol production.

In the sample with sediment 1, there are two crystalline phases of Na_2_HPO_4_ ∙ 2H_2_O (ICSD 01-074-6210) with characteristic reflections for the angle 2θ: 10.5°; 16.8°; 19.1°; 27.3°; 31.1°; 32.6°, and KH_2_PO_4_ phase (LPF 04-007-5247) with reflections for 2θ angles: 23.9°; 30.7°; 33.9°; 46.5°. No other crystalline phases have been found, and if they are present, they are of small content in relation to those found. The Na_2_HPO_4_ ∙ 2H_2_O phase is approximately 62%, and KH_2_PO_4_ is approximately 38% of the crystal phases assigned to the performed diffraction pattern (Figure 2).

**Figure 3:**
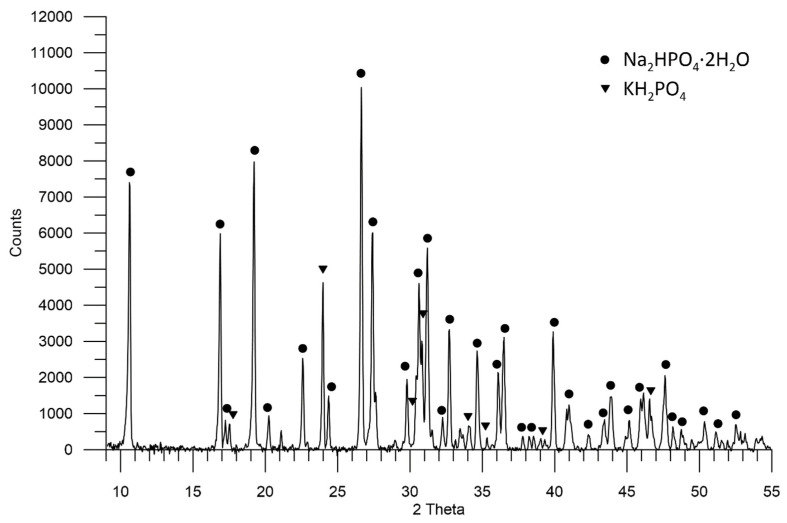
A diffractogram showing the composition of sediment 2 in the sample of waste phosphate from polyol production.

In the sample with sediment 2, there are two crystalline phases of Na_2_HPO_4_ ∙ 2H_2_O (ICSD 01-074-6210) with characteristic reflections for the angle 2θ: 10.5°; 16.8°; 19.1°; 27.3°; 31.1°; 32.6° and KH_2_PO_4_ phase (LPF 04-007-5247) with reflections for 2θ angles: 23.9°; 30.7°; 33.9°; 46.5°. No other crystalline phases have been found, and if they are present, they are of small content in relation to those found. The Na_2_HPO_4_ ∙ 2H_2_O phase is estimated to be 92%, and KH_2_PO_4_ to be approx. 8% of the crystal phases assigned to the performed diffraction pattern (Figure 3).

**Figure 4:**
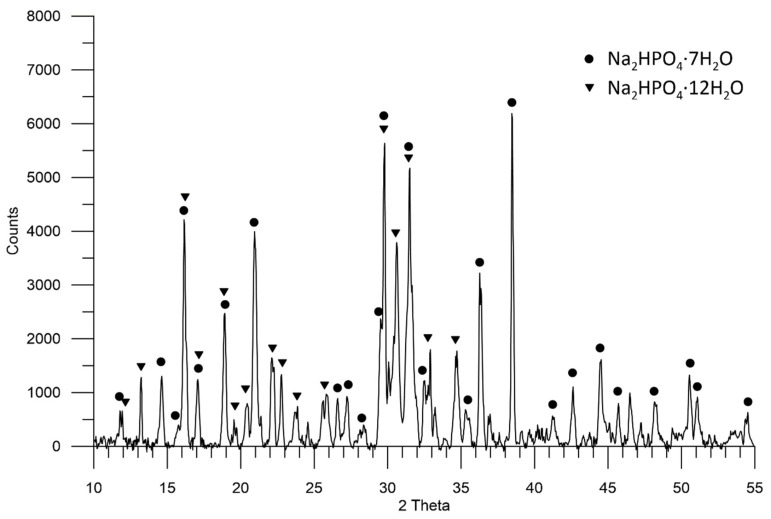
A diffractogram showing the composition of sediment 3 in the sample of waste phosphate from polyol production.

In the sample with sediment 3, the crystalline phase of Na_2_HPO_4_ ∙ 7H_2_O (ICDD: 00-010-0191) with characteristic reflections at the angle of 2θ: 16.2° can be distinguished: 20.9°; 29.5°; 31.2°; 31.7°. The second crystalline phase is Na_2_HPO_4_ ∙ 12H_2_O (ICDD 00-011-0657) with characteristic reflections at the angle of 2θ: 14.6°; 16.2°; 17.1°; 20.5°; 22.2°; 22.7°; 29.8°; 31.4°; 36.3°. These are the main crystallographic phases recorded on the diffraction pattern. Due to the lack of relevant information in the database for this sample, it is not possible to estimate the content of the phases present in it (Figure 4).

Sediment 1, which is the finest crystal, consists of two phases: hydrated secondary alkali metal phosphate—Na_2_HPO_4_ · 2H_2_O (~62%) and primary alkali metal phosphate—KH_2_PO_4_ (~38%). On the other hand, sediment 2, whose crystals are larger, mainly consists of Na_2_HPO_4_ · 2 H_2_O (~92%). The largest crystals are formed by hydrated secondary phosphates: Na_2_HPO_4_ ∙ 7H_2_O and Na_2_HPO_4_ ∙ 12H_2_O in sediment 3. They pose the greatest threat during fertilizer application. The conditions for the formation of individual layers in the waste phosphate sludge require further studies.

## 3. Materials and Methods

The research material is waste sodium–potassium phosphate from PCC Rokita S.A. in Brzeg-Dolny, Poland. There, waste phosphates are formed in the polyol production plant in the phase separation stage, where the organic phase (product), the aqueous phosphate phase, and the interphase containing the filter aid are separated. Phosphates are formed as a result of the neutralization of sodium and potassium ions (from the polymerization catalyst) with acidic disodium pyrophosphate. 

Waste phosphates are in the form of an aqueous suspension with a water content of 49%. At PCC Rokita, it is not possible to reuse them in the polyol production process. However, the phosphorus salts contained in them are well-absorbed by plants and can be successfully used as a fertilizer component. The high water content of the waste, which is peeled after the production of polyols, significantly limits the possibilities of its economic processing. Evaporating such an amount of water would be very energy-consuming, which is associated with high costs. The best solution is to use unchanged waste phosphorus salts for the production of liquid suspension fertilizers.

### 3.1. Methods for the Analysis of Organic Carbon Content

In order to determine the feasibility of producing a given type of fertilizer, it is necessary to know the content of carbon in its organic form. According to the new EU regulation 2019/1009 of 5 June 2019, the organic carbon content classifies the fertilizer into the appropriate category (organic, organo-mineral, or mineral fertilizer). Belonging to particular categories of fertilizers determines further quantitative and qualitative requirements [24].

The organic carbon content was analyzed using the InnovOx series TOC analyzer using the supercritical water oxidation technology. CO_2_ determination is performed using NDIR spectrometry. First, the total inorganic carbon (TIC) in the sample is determined by acidification (lowering the pH turns carbonates and bicarbonates into CO_2_). This carbon dioxide is blown from the sample and then directed either to the atmosphere (NPOC measurement) or to an NDIR detector (TOC measurement), which calculates the carbon dioxide content based on specific wavelength absorption. After detection, the device calculates the TIC and reports as parts per million (ppm) of the TIC. The NDIR detector allows one to determine the amount of carbon regardless of changes in sample pH or temperature and prevents the potential influence of other gases such as chlorides, chlorine dioxide, sulfur dioxide, etc., on the result. The analyzer measures total organic carbon (TOC) by measuring carbon dioxide obtained from chemical oxidation of organic carbon in the sample. After removing TIC from the sample, the analyzer doses sodium persulfate (Na_2_S_2_O_8_), which, as a strong oxidant, quickly reacts with organic carbon in the sample (at approx. 100 °C) to form carbon dioxide. After the oxidation process is complete, carbon dioxide is blown from the sample and determined on the NDIR detector. When the detector calculates the TOC content of the sample, it generates a result in ppm TOC.

### 3.2. Methods of Chemical Analysis of Waste

The key issue determining the suitability of waste for fertilization purposes is the content of plant nutrients and the level of impurities. Therefore, the content of the expected nutrients in the waste was determined: phosphorus (P), potassium (K), sodium (Na) and impurities in the form of: cadmium (Cd), chromium (Cr VI), mercury (Hg), nickel (Ni), lead (Pb), arsenic (As), copper (Cu), zinc (Zn), iron (Fe), and aluminum (Al.). The standard methods and procedures listed in Table 4 were used for the tests.

### 3.3. Methods of Sludge-in-Waste Analysis

**Figure 5:**
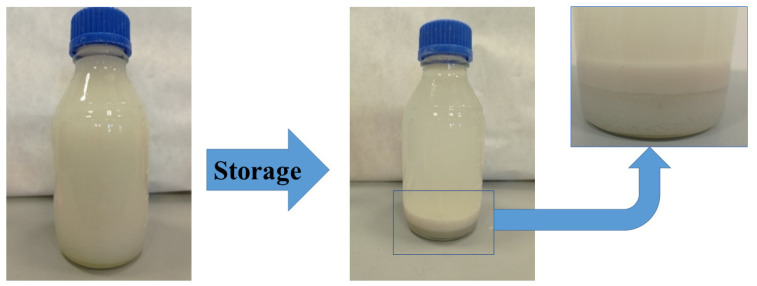
Waste phosphate immediately after mixing with compressed air (**left**) and after one day of storage (**right**).

Waste sodium–potassium phosphate is a white suspension that easily delaminates during storage (Figure 5). 

There are three sediment layers with different crystal sizes. The sediments from individual layers were in a semi-liquid form and were separated by decantation. In order to avoid mixing the crystals from the individual layers, the sediment at the junction of the layers was poured separately and not used for further studies. The individual layers of the sediment were then dried and subjected to X-ray fluorescence analysis (XRF) to determine their composition. XRD measurements were carried out on a PANalytical Empyrean powder diffractometer operating in the Bragg–Brentano geometry. The standard equipment includes a Cu X-ray tube, a rotating transmission-reflection table and a fast linear PIXcel3D detector. The intensity and voltage of the X-ray tube were respectively: I = 40 mA and U = 40 kV. PreFix system configuration (pre-aligned fast interchangeable X-ray modules) made it possible to carry out measurements with the following optical beam settings:

Optical path parameters for the XRD incident beam:

- 0.04 rad soller gap;
- mask: 5 mm;
- slit for beam divergence adjustment: 1/2⁰;
- anti-scatter gap: 1⁰.

Optical path parameters for the XRD diffracted beam:

- 0.04 rad soller gap;
- anti-scatter gap: 8 mm PIXel 3D;
- elliptical mirror.

XRD Scan Parameters:

- initial angle [⁰2θ]: 5;
- end angle [⁰2θ]: 75;
- scan step [⁰2θ]: 0.0394;
- exposure time [s]: 68.6;
- sample rotation: yes, disc rotation with a period of 4 s.

The material was ground in a mortar (including the obtained crystals) and placed on a table enabling work in a reflection system with the possibility of sample rotation. All measurements were made on the same program with the use of the same incident beam and diffracted beam optics for the entire series. The diffractogram analysis was performed in the HighScorePlus program. The quantitative analysis of the crystallographic phases was performed automatically with the Rietveld method with manual refinement of the results to obtain the best matching parameters. Rietveld’s analysis consists of refining the parameters of the crystal structure by applying the least squares method, fitting the theoretical curve to experimental data. However, the application of this method is usually limited to well-characterized phases in which the number of structural defects is limited. The quantitative determination of the phase content using the Rietveld method can be problematic, and the numerically calculated (using a crystal model with ideal parameters) X-ray diffraction signal can significantly differ from the recorded signal. The problem may also be the uptake by the grain of chemical compounds included in the samples of privileged orientation, which disturbs the quantitative ratios of the crystalline phases read from the diffractogram. In the case of tested samples, the quantitative analysis can only be used as a guide.

## 4. Conclusions

The conducted research confirms that the analyzed waste does not contain substances harmful to plants. However, it contains significant amounts of phosphorus, which makes it an attractive raw material for the production of fertilizers. The waste does not require treatment or other preliminary preparation processes and, unchanged, can be used in the production of suspension fertilizers. As a result, the phosphorus it contains can be fully recovered and reused in fertilization. Its use is in line with the EU’s circular economy strategy and meets the challenges posed by the new fertilizer regulations contained in the EU Regulation 2019/1009.

Further research should be aimed at checking the possibility of enriching waste phosphates with additional nutrients: macro- and micronutrients. In addition, the research should cover methods of stabilizing the produced fertilizer suspensions and the conditions of their storage, preventing the formation of sediment with large crystals.

## Figures and Tables

**Table 1:** TOC analysis results for waste samples collected on the surface.

Sample	TOC [ppm]	Average
1	137	151
	137	
	178	
2	246	276
	239	
	344	
3	457	461
	460	
	467	
4	383	383
	372	
	394	
5	445	419
	436	
	377	
6	318	355
	344	
	402	
Average	341	

**Table 2:** TOC analysis results for mixed waste samples.

Sample	TOC [ppm]	Average
1	555	574
	628	
	572	
2	592	581
	616	
	557	
3	555	585
	628	
	572	
Average	586	

**Table 3:** The results of the chemical analysis of the waste.

Tested Feature	Unit	Result y ± U ^1^	Requirements for Suspension Fertilizers According to EU Regulation No. 2019/1009
Phosphorus soluble in mineral acids expressed as P_2_O_5_	%	23.46 ± 0.3	min. 1.5% by mass of total phosphorus pentoxide (P_2_O_5_)
Potassium as K_2_O	%	9.89 ± 0.16	min. 1.5% by mass of total potassium oxide (K_2_O)
Sodium (Na)	%	7.60	min. 0.5%-max. 20% by mass of total sodium oxide (Na_2_O)
Copper (Cu)	mg/kg	less than 1.0	6002
Iron (Fe)	mg/kg	46.0	-
Zinc (Zn)	mg/kg	less than 1.0	15002
Cadmium (Cd)	mg/kg	less than 1.0	60
Lead (Pb)	mg/kg	less than 8.0	120
Nickel (Ni)	mg/kg	less than 1.0	100
Aluminum (Al)	mg/kg	74.7	-
Arsen (As)	mg/kg	less than 4.0	40
Mercury (Hg)	mg/kg	less than 0.002	1
Chrome (VI)	mg/kg	less than 0.3	2

^1^ The reported expanded uncertainty (U) is based on the standard uncertainty multiplied by a coverage factor k = 2 providing a confidence level of 95%. The uncertainty of sampling was not taken into account in the calculations (U). These limit values do not apply where an ingredient has been intentionally added to correct deficiencies in soil.

**Table 4:** Research methods used for waste analysis.

Tested Feature	Test Method	Procedure
Phosphorus soluble in mineral acids expressed as P_2_O_5_	weight method	PN-EN 15956:2011 PN-EN 15959:2011
Potassium expressed as K_2_O	weight method	PN-EN 15477:2009
Sodium (Na) Copper (Cu) Iron (Fe) Zinc (Zn) Cadmium (Cd) Lead (Pb) Nickel (Ni) Aluminum (Al)	inductively coupled plasma atomic emission spectrometry (ICP-OES)	PB 35 ed. III of 02/03/2020 PN-EN 16319 + A1: 2016-02 with the exception of point 8.2
Arsen (As)	inductively coupled plasma atomic emission spectrometry (ICP-OES)	PB 35 ed. II of 02/03/2020 PN-EN 16317 + A1: 2017-04 with the exception of point 8.2
Mercury (Hg)	atomic absorption spectrometry with the amalgamation technique	RMG annex 3, p. 4 *
Chrome (VI)	ion chromatography	PN-EN 16318+A1:2016-03

* RMG—Ordinance of the Minister of Economy of 8 September 2010 on the method of packaging mineral fertilizers, placing information on fertilizer ingredients on these packages, methods of testing mineral fertilizers and types of fertilizer lime. (Journal of Laws No. 183 item 1229).

## Data Availability

The data presented in this study are available on request from the corresponding author.

## Sample Availability

Samples of the compounds are not available from the authors.
